# An information-motivation-behavioural skills analysis of long-lasting insecticidal net use among pregnant women in a hospital in North-Eastern Nigeria

**DOI:** 10.1186/s12874-019-0803-z

**Published:** 2019-07-18

**Authors:** Ahmed Dahiru Balami, Salmiah Md Said, Nor Afiah Mohd Zulkefli, Norsa’adah Bachok, Emmanuel Luke Balami

**Affiliations:** 1 Department of Community Health, Faculty of Medicine and Health Sciences, Universiti Putra Malaysia, Selangor Darul Ehsan, Malaysia; 2 Unit of Biostatistics and Research Methodology, Universiti Sains Malaysia, Kelantan, Malaysia; 3 Innovative Management Consultant, Maiduguri, Borno State Nigeria

**Keywords:** Information-motivation-behavioural skills (IMB) model, Behaviour, Long-lasting insecticidal net (LLIN), Pregnant women

## Abstract

**Background:**

Sleeping under a long-lasting insecticidal net (LLIN) is recommended for all pregnant women in sub-Saharan Africa, due to the high prevalence of malaria infection and its associated complications in the region. Despite this, LLIN use has still remained sub-optimal among pregnant women in Maiduguri, Nigeria. Understanding the interplay of factors influencing this important health behaviour would guide the development of interventions to promote its adoption.

**Methods:**

Data was collected from 380 randomly selected antenatal care attendees of a hospital in Maiduguri, using structured questionnaires. This data was then used to test the Information-Motivation-Behavioural Skills (IMB) model, for model fit, and interrelations among the constructs, using the structural equation modelling analysis with Smart-PLS.

**Results:**

Information and motivation were significantly related to behavioural skills (*r* = 0.29, *p* < 0.001 and *r* = 0.37, *p* < 0.001, respectively); and also to behaviour (*r* = 0.22, *p* < 0.001 and *r* = 0.11, *p* = 0.033 respectively). Behavioural skills however, did not significantly relate to behaviour (*r* = 0.03, *p* = 0.278).

**Conclusion:**

These findings highlight the potential usefulness of the IMB model in guiding interventions for promoting LLIN use among this group. More emphasis should also be laid on boosting levels of information and motivation among the target group.

**Electronic supplementary material:**

The online version of this article (10.1186/s12874-019-0803-z) contains supplementary material, which is available to authorized users.

## Background

Malaria remains a disease of public health importance in sub-Saharan Africa, with Nigeria contributing the most number of cases to its global incidence [1]. Malaria during pregnancy is associated with several complications like: maternal anaemia [2–6], low-birth weight [7, 8], preterm delivery [9, 10], abortion [11, 12] and still birth [13, 14]. Pregnant women have been reported to have higher risks of contracting malaria [15, 16], and also attract twice more mosquitoes than their non-pregnant counterparts [17]. In Borno state, Nigeria, malaria prevalence as high as 60.3% [18] and 44.5% [19] have been reported among ante-natal care attendees of a tertiary health centre. Regularly sleeping under a long-lasting insecticidal net (LLIN) significantly reduces the incidence of malaria and its complications during pregnancy [20, 21]. The World Health Organization (WHO) as such, recommends its use, to all pregnant women in malaria-endemic areas of sub-Saharan Africa [22]. Despite these recommendations, compliance with LLIN has remained very low, as reported in the National Health and Demographic Survey (NHDS) that only 13.8% of pregnant women in Borno state had slept under an LLIN the night before the survey [23]. In Urban Borno, 11.3% of households had at least one LLIN for every two persons, while 55.5% of pregnant women in north-eastern Nigeria had an LLIN [24]. Even in a tertiary health centre in Maiduguri, only 2.8% of its antenatal care attendees were sleeping under an LLIN [25].

**Fig. 1:**
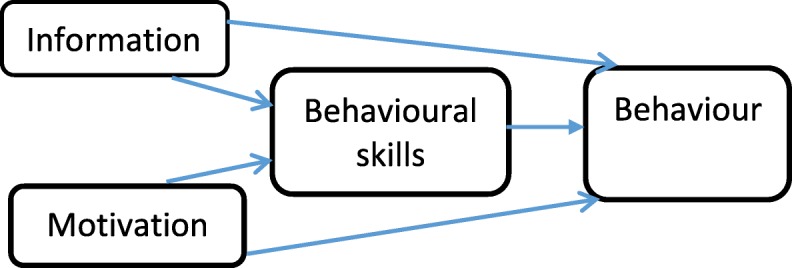
The Information-Motivation-Behavioural Skills Model. It focuses on three components which result in a behavioural change, which are: relevant information on the desired behaviour; the second is appropriate motivation to carry out such behaviours, while the third is ensuring that the individual is equipped with the necessary skills and competencies to carry out the desired behaviours

Understanding the interplay of determinant factors for LLIN use among pregnant women could guide the development of health interventions to promote its adoption. The Information-Motivation-Behavioural skills (IMB) theory has proven to be a useful model in explaining the pathway to some health behaviours like HIV preventive behaviour [26], diabetes self-care [27], and even curb-side recycling behaviour [28]. A health educational intervention guided by the model was also effective in improving HIV preventive behaviours among truck drivers in India [29]. This model was first suggested by Fisher and Fisher, to explain HIV preventive behaviours among college students [26]. It captures the psychological determinants of performing health behaviours which have an impact on health. The model, as illustrated in Fig. 1, emphasizes that information about a health behaviour, even though necessary, is not enough to cause a behavioural change [30]. It also underscores the need for high levels of motivation, which comprises both personal and social motivation [31]. Behavioural skills on the other hand, entail both the actual as well as perceived abilities to carry out the desired health behaviour [31].

The main objective of the study was to adapt the IMB model to analyze LLIN use among pregnant women, with the following specific objectives:

- To determine the relationship between ‘information’ and ‘behavioural skills’ among the respondents.
- To determine the relationship between ‘motivation’ and ‘behavioural skills’ among the respondents.
- To determine the relationship between ‘behavioural skills’ and ‘behaviour’ among the respondents.
- To determine the relationship between ‘information’ and ‘behaviour’ among the respondents.
- To determine the relationship between ‘motivation’ and ‘behaviour’ among the respondents.
- To determine the mediating effect of ‘behavioural skills’ on the relationship between ‘information’ and ‘behaviour’ among the respondents.
- To determine the mediating effect of ‘behavioural skills’ on the relationship between ‘motivation’ and ‘behaviour’ among the respondents.

## Methods

### Study location

The study area was Maiduguri, the Borno state capital in north-eastern Nigeria. Its vegetation is Sudan savannah, with a climate that varies according to the time of the year, with temperatures ranging from 25 °C to 44 °C, and an average annual rainfall of 613 mm [32]. It has a population of 540,016, consisting of 282,409 males and 257,607 females [33]. Maiduguri hosts 432,785 persons displaced from various local government areas of Borno state, by the Boko Haram insurgency [34]. The main economic activities are agriculture and trading, while Hausa and Kanuri are the main languages spoken [32]. The study was conducted at the State Specialist Hospital, Maiduguri (SSHM), which is centrally-located in Maiduguri. The hospital’s ANC clinic receives an average of about 100 clients every day, and which is the highest in the state. A previous study had revealed a malaria prevalence of 48.1% among the hospital’s antenatal care attendees [35]. LLINs are not provided at the hospital.

### Study design and participants

This cross-sectional study was conducted among antenatal care (ANC) attendees of the SSHM. The criteria for inclusion into the study was to be a pregnant woman registered for her first ANC at the SSHM. As this study formed the baseline of a health educational intervention study, those with conditions that could influence the outcome variables were excluded. ANC attendees who were not resident in Maiduguri, those with hypertension, and/or diabetes mellitus were excluded, as these conditions could affect pregnancy outcomes [36–38]. Given power of 80% with 0.05 level of significance, four latent variables and 27 observed variables, the A-priori Sample Size Calculator for Structural Equation Models [39] was used to calculate the minimum sample size required to detect effect – 137, and model structure – 341. Three hundred and eighty respondents were selected from eight ANC booking sessions through a systematic random sampling, from 30 January to 20 March, 2017.

### Instrument and data collection

A structured questionnaire (Additional file 1) comprising of five sections: socio-demography, information, motivation, behavioural skills and behaviour, was used to collect data from the respondents. Items for the information construct were adopted from the instruments used in some previous studies [40–42], after obtaining permission from the authors. This section had 18 questions, each with three options: Yes, No and I don’t know. A correct response was scored one (1), while an incorrect response was scored zero (0). The sum total for information scores which had a possible range from zero (0) to 18 points was then computed, and used for the analysis. Items for motivation and behavioural skills were adapted from the items of respective sections of a previous study [27]. Three items were used to assess motivation, the first two, asking of the level of goodness for their health and the level of pleasantness of sleeping under an insecticidal net, with Likert responses which ranged from 1 = very bad to 5 = very good; and 1 = very unpleasant to 5 = very pleasant respectively. The third was: ‘Most people who are important to me think I should sleep more frequently under an insecticidal net’, with Likert responses ranging from 1 = very untrue to 6 = very true. For behavioural skills, there were five questions: The first asked of how easy or hard it would be to sleep under an insecticidal net every night’ with Likert responses ranging from 1 = very hard to 4 = very easy. The other four questions asked of how effectively they could hang an insecticidal net, sleep more frequently under it, check for and repair rifts in it, and persuade others to support their sleeping under it. These had Likert responses ranging from 1 = very ineffectively to 4 = very effectively. Behaviour was measured as frequency of insecticidal net use per week, which was categorized as: Never, Seldom (1–2 times a week), Sometimes (3–4 times a week), Often (5–6 times a week) and Almost always. These categories were scored, 1, 2, 3, 4 and 5 respectively.

The questionnaire was first developed in English language and then forwardly translated to Hausa language by a Senior University staff of the linguistics department. A back translation of the Hausa version to English, was done afterwards by a different scholar of similar qualification. The original English version and the back translated English version were then compared by a Public Health Specialists, who was not part of the researchers. A pilot study of the Hausa version was then conducted with a sample of 190 respondents, and the sections for motivation and self-efficacy had Cronbach’s alpha values of 0.87 and 0.77 respectively. Sixty three out of the 190 initial respondents were made to fill the questionnaire again after two weeks to determine its reliability. All items for the information, motivation, and behavioural skills sections had Cohen’s kappa scores above 0.7. Owing to low literacy rates among females in Maiduguri [43], enumerators were used to collect the data via face-to-face interviews, using the questionnaire. All the five enumerators were holders of Diploma certificates in Public Health.

Due to low literacy levels, informed verbal consent was obtained from each respondent after they had been taken through the respondent information sheet. No additional consent was obtained from the husbands or guardians of those below 18 years, as they were considered emancipated minors, since they were married women. The study protocol, as well as methods of obtaining consent, had been approved by the Ethics Committee of the State Specialist Hospital Maiduguri (SSH/GEN/64/Vol.1) and the Ethics Committee for Research Involving Human Subjects of the Universiti Putra Malaysia (UPM/TNCPI/RMC/1.4.18.2) before the study commenced.

### Analysis

The data obtained was entered into Microsoft Excel spreadsheet (Additional file 2), followed by data cleaning. Frequency and percentage were used to describe the socio-demographic characteristics, and Chi-squared tests were conducted to determine the association between these factors and frequency of insecticidal net use. The research variables were not normally distributed, and as such, PLS-SEM approach was used to assess the relationship between the constructs in the IMB model. In the model, information and motivation were considered as exogenous variables, behaviour as endogenous, while behavioural skills was considered both endogenous and exogenous. The measurement model was initially evaluated to assess the measurement properties of the observed variables, after which the structural model was evaluated.

## Results

The respondents’ ages ranged from 15 to 45 years, with mean (SD) age of 26.5 (5.8) years. As presented in Table 1, those of Kanuri ethnicity were the highest in number (35.8%); most were also permanent residents of Maiduguri (73.4%), with 58.9% having some level of education. Only a few (7.6%) did not earn less than the N18,000 Nigerian minimum wage. Table 2 shows that there was no significant association between any of the socio-demographic factors studied and frequency of insecticidal net use.

**Table 1:** Respondents’ socio-demographic characteristics (*N* = 380)

Socio-demographic characteristics	Frequency	Percentage (%)
Age group (years)		
26 and below	208	54.7
27 and above	172	45.3
Total	380	(100.0)
Ethnicity		
Kanuri	136	(35.8)
Hausa	59	(15.5)
Babur	33	(8.7)
Fulani	39	(10.3)
Others	113	(29.7)
Total	380	(100.0)
Education		
None	156	(41.1)
Primary	67	(17.6)
Secondary	108	(28.4)
Tertiary	49	(12.9)
Total	380	(100.0)
Occupation status		
Employed	172	(45.3)
Not employed	208	(54.7)
Total	380	(100.0)
Income level		
Below minimum wage	351	(92.4)
At/above minimum wage	29	(7.6)
Total	380	(100.0)
Type of residence		
Permanent resident	279	(73.4)
Internally displaced	101	(26.6)
Total	380	(100.0)

**Table 2:** Association between socio-demographic factors and frequency of ITN use

Variables	Age group				*χ* ^*2*^	*df*	*p*
	26 years and below		27 years and above				
Freq. ITN use					2.872	4	0.579
Never	123	(59.1)	95	(55.2)			
Seldom	17	(8.2)	12	(7.0)			
Sometimes	16	(7.7)	20	(11.6)			
Often	24	(11.5)	25	(14.5)			
Almost always	28	(13.5)	20	(11.6)			
	Ethnicity						
	Kanuri		Others				
Freq. ITN use					4.165	4	0.384
Never	80	(58.8)	138	(56.6)			
Seldom	7	(5.1)	22	(9.0)			
Sometimes	15	(11.0)	21	(8.6)			
Often	14	(10.3)	35	(14.3)			
Almost always	20	(14.7)	28	(11.5)			
	Education						
	None		Educated				
Freq. ITN use					2.559	4	0.634
Never	90	(57.7)	128	(57.1)			
Seldom	8	(5.1)	21	(9.4)			
Sometimes	16	(10.3)	20	(8.90			
Often	21	(13.5)	28	(12.5)			
Almost always	21	(13.5)	27	(12.1)			
	Employment status						
	Unemployed		Employed				
Freq. ITN use					7.458	4	0.114
Never	126	(59.4)	92	(54.8)			
Seldom	17	(8.0)	12	(7.1)			
Sometimes	25	(11.8)	11	96.5)			
Often	22	(10.4)	27	(16.1)			
Almost always	22	(10.4)	26	(15.5)			
	Income level						
	Below min. Wage		At/above min wage				
Freq. ITN use					2.669	4	0.615
Never	203	(57.8)	15	(51.7)			
Seldom	26	(7.4)	3	(10.3)			
Sometimes	35	(10.0)	1	(3.4)			
Often	44	(12.5)	5	(17.2)			
Almost always	43	(12.3)	5	(17.2)			
	Residence type						
	Permanent resident		IDP				
Freq. ITN use					6.297	4	0.178
Never	157	(56.3)	61	(60.4)			
Seldom	26	(9.3)	3	(3.0)			
Sometimes	29	(10.4)	7	(6.9)			
Often	35	(12.5)	14	(13.9)			
Almost always	35	(12.5)	14	(13.9)			

The results of the measurement model are presented in Table 3. The outer loadings ranged from 0.532 to 0.843, which were all above the minimum acceptable threshold of 0.5 [44]. In addition, the Cronbach’s alpha values (0.784 and 0.79) and Composite reliability coefficients (0.872 and 0.857) of Motivation and Behavioural skills were considered acceptable. The variance inflation factors (VIFs) also ranged from 1 to 2.115, as shown in Table 4, indicating there was no multi-collinearity, since none was above 5.

**Table 3:** Reliability and validity of constructs

Construct	Items	Outer Loading	Cronbach’s Alpha	Composite Reliability	Average Variance Extracted (AVE)
Behavioural Skills	B_SKILLS_1	0.532	0.79	0.857	0.553
	B_SKILLS_2	0.654			
	B_SKILLS_3	0.817			
	B_SKILLS_4	0.828			
	B_SKILLS_5	0.837			
Motivation	MOT1	0.829	0.784	0.872	0.694
	MOT2	0.826			
	MOT3	0.843			
Information	TOTAL_INFO	1	1	1	1
Behaviour	BEHAVIOUR	1	1	1	1

**Table 4:** Variance inflation factor

Behaviour	1
B_SKILLS_1	1.151
B_SKILLS_2	1.482
B_SKILLS_3	1.984
B_SKILLS_4	2.038
B_SKILLS_5	2.115
MOT1	1.875
MOT2	1.816
MOT3	1.436
Total_Info	1

**Fig. 2:**
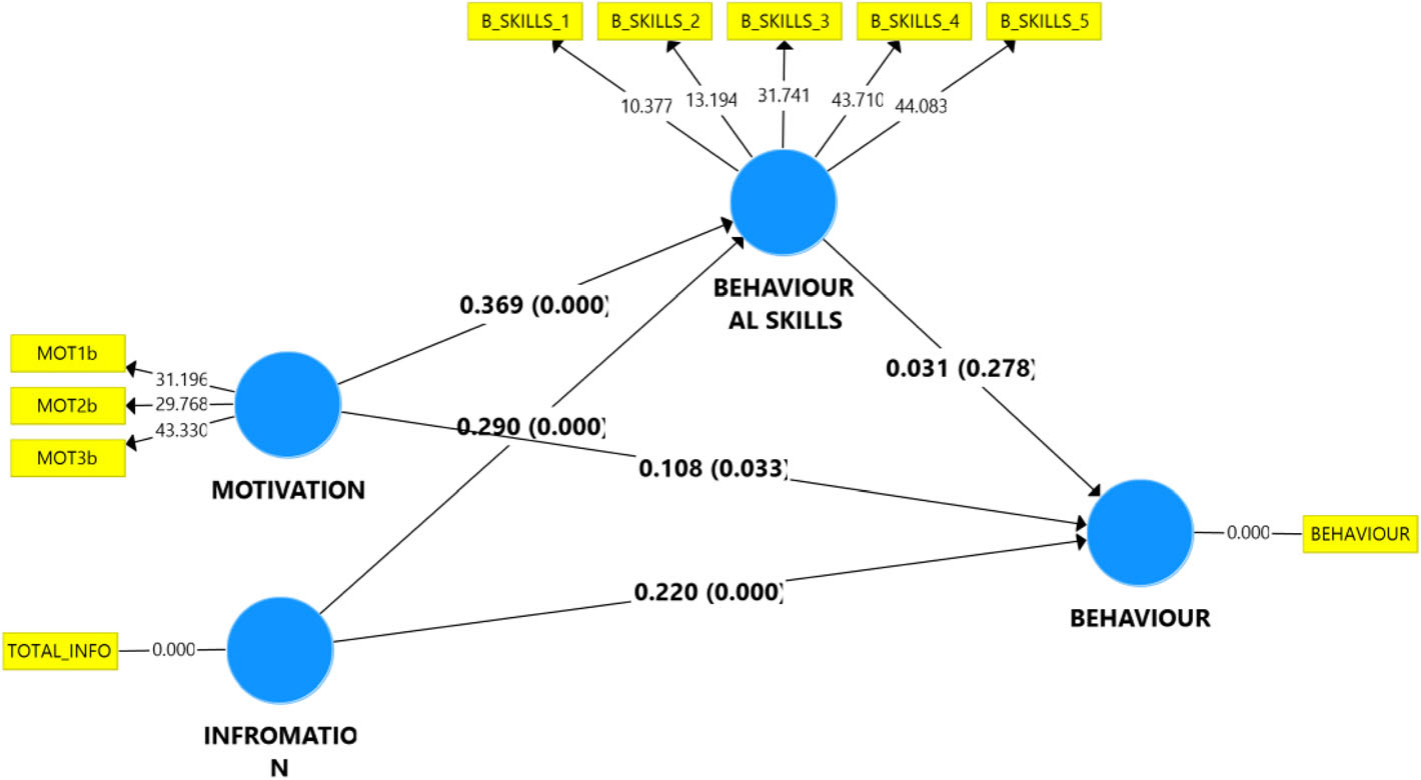
Path model of the IMB constructs

The path model between the constructs is shown in Fig. 2, and the results of the relationship between variables is also presented in Table 5. All the variables correlated significantly with each other, except for behavioural skills, which was not significantly correlated with behaviour (B = 0.031, *p* = 0.278). The strongest correlation was between motivation and behavioural skills (B = 0.369, *p* = < 0.001). Results from Table 6 also indicate that the respective indirect paths of information and motivation via behavioural skills were not significant.

**Table 5:** Path coefficients and hypothesis testing: direct effect

	Original Sample (O)	Sample Mean (M)	Standard Deviation	T Statistic (|O/STDEV|)	*P*-value
Infromation - > Behaviour	0.22	0.219	0.049	4.522	< 0.001
Motivation - > Behaviour	0.108	0.109	0.059	1.839	0.033
Infromation - > Behavioural skills	0.29	0.29	0.048	6.083	< 0.001
Motivation - > Behavioural skills	0.369	0.372	0.048	7.673	< 0.001
Behavioural skills - > Behaviour	0.031	0.031	0.053	0.589	0.278

**Table 6:** Path coefficients and hypothesis testing: indirect effect

	Original Sample (O)	Sample Mean (M)	Standard Deviation (STDEV)	T Statistics (|O/STDEV|)	*P* values
Infromation - > Behaviour	0.009	0.009	0.016	0.58	0.281
Motivation - > Behaviour	0.011	0.011	0.02	0.576	0.282

## Discussion

The structural model, as presented in Fig. 2 and Table 5, shows that having more information on malaria, its complications, the safety and effectiveness of insecticidal nets (*r* = 0.222; *p* < 0.001), as well as strong motivation for sleeping under an insecticidal net (*r* = 0.108; *p* = 0.033), are likely to be the main determinants of insecticidal net use among this group. This is in line with findings from a previous study on curb side recycling behaviour, where information and motivation showed a similar relationship with behaviour [28]. In previous studies, knowledge of malaria [45] and insecticidal net [46] were important determinants of insecticidal net use. Similarly, belief that insecticidal nets provide protection against malaria, was significantly associated with its use [47, 48]. R^2^ being less than 10% (0.083), suggests that there could be other important factors responsible for predicting of insecticidal net use behaviour among the study group. Information and motivation constructs however, played a significant role in predicting behavioural skills among the respondents, with behavioural skills R^2^ being about 30% (0.281). This agrees with the evaluation of the predictive relevance (Q^2^), where the two exogenous constructs (Information & Motivation) were more relevant in predicting behavioural skills (0.142), than the three constructs (Information, Motivation & Behavioural Skill) in predicting behaviour (0.07). However, in determining the effect size (f^2^), the information construct had greater effect in determining behaviour (0.044), while motivation had more effect in determining behavioural skill (0.174).

Conversely, in determining the total effect of the driver constructs on the target constructs, the motivation construct had higher total effect (0.119) than both the information and behavioural skills constructs (0.009 and 0.031 respectively) on the behaviour construct. Similarly, the motivation construct had higher total effect (0.369) on the behavioural skills construct than the information construct (0.290). This shows that interventions aimed at promoting LLIN use should lay much emphasis on increasing motivation levels. By also taking the construct’s indicator outer loadings into consideration, we identified the specific element of the motivation construct which needs to be addressed, which was MOT3. This item had the highest outer loading (43.33), and it asked of how true it was, that the people most important to them thought they should sleep more frequently under an insecticidal net. This highlights the important role of significant others, in influencing health behaviour. This finding can be validated, as these respondents (pregnant women) live in a collective/culture distance society, where respect is given to some selected individuals as against individualistic societies. Even a systematic review had identified household decision as an important determinant of ITN use among pregnant women in Africa [48]. As such, health promotion interventions on ITN use should seek to reach out to these important persons too.

The structural model also shows that behavioural skills is neither a mediating factor between information and behaviour, nor between motivation and behaviour. When the items of the behavioural skills component are related to the requirements of using an ITN, it could be clear why behavioural skills did not play a significant role among this sample. Firstly, inability to properly hang or care for an LLIN may not be sufficient to prevent one from sleeping under it, as these procedures could be done by someone else, especially in northern Nigeria where pregnant women are generally considered delicate and usually have some female relative(s) or friend(s) to care for them during their pregnancies. For such women, sleeping under an LLIN would basically be dependent on their access to one, and their choice to sleep under it. This point is further buttressed by the fact that social support from significant others played a very significant role in determining those who slept under an insecticidal net. The role of behavioural skills in influencing health behaviour is likely dependent on the cultural setting in question, as contrasting results were found in a study among pregnant women in Congo, where self-efficacy, a component of behavioural skills, was a significant determinant of insecticidal net use [48].

A major limitation of the IMB model is its failure to account for environmental and cultural factors [31]. As such, important determinants of LLIN use like access to LLIN [40, 49, 50], socio-economic status, parity [51], educational level [52], and monthly income [53] were not included in the IMB model analysis. However, some of these important variables like age group, ethnicity, educational status, occupational status, income level, and residence type, had shown no significant association with LLIN use in this study.

## Conclusions

This study reveals the significant roles information on malaria and LLIN, as well as motivation for LLIN use play in influencing LLIN use among pregnant women. It is therefore recommended that the design of health education modules on malaria prevention be guided by the IMB model, while laying much emphasis on information and motivation.

## Additional files

Additional file 1: **Study Questionnaire.** The Hausa version questionnaire used to collect data for this study and the English language translation. (DOCX 143 kb)

Additional file 2: **Study Dataset.** Spreadsheet of the raw data collected and analyzed in this study. To maintain respondents’ anonymity, only two indirect identifiers were retained (age and residence type). (XLSX 46 kb)

## Data Availability

The questionnaire and raw data set for this study are available as additional files.

## Abbreviations

ANC: Antenatal care
IMB: Information-motivation-behavioural skills
LLIN: Long-lasting insecticidal net
NHDS: National Health and Demographic Survey
PLS-SEM: Partial least squares Structural equation modelling
WHO: World Health Organization
