# Supplementary material for: An information-motivation-behavioural skills analysis of long-lasting insecticidal net use among pregnant women in a hospital in North-Eastern Nigeria
Source: BMC Med Res Methodol. 2019 Jul 18;19:157. doi: 10.1186/s12874-019-0803-z (PMC6637635; doi:10.1186/s12874-019-0803-z)
Supplement: Supplementary file 1 — Study Questionnaire. The Hausa version questionnaire used to collect data for this study and the English language translation. (DOCX 143 kb) [file 12874_2019_803_MOESM1_ESM.docx]

**QUESTIONNAIRE**

| **ID NO:** |  |  |  |  |  |  |  |  |
| --- | --- | --- | --- | --- | --- | --- | --- | --- |


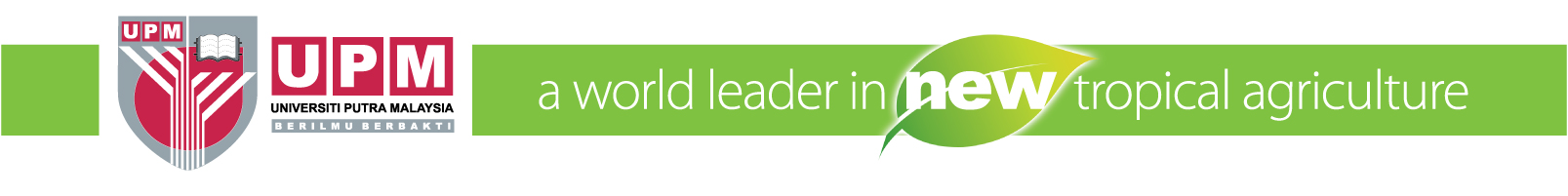


**EFFECTS OF A HEALTH EDUCATIONAL INTERVENTION ON MALARIA**

**PREVENTIVE BEHAVIOUR AND ADVERSE PREGNANCY OUTCOMES AMONG**

**PREGNANT WOMEN IN A SECONDARY-LEVEL HEALTH CENTRE IN**

**MAIDUGURI, BORNO STATE, NIGERIA**

**RESEARCHER: AHMED DAHIRU BALAMI**

**(PhD Student, Department of Community Health, Universiti Putra Malaysia)**

**Instructions:** Please answer ALL questions and please TICK (√) only one answer for each statement, in the appropriate box.

**Reminder:** Please try to answer all questions honestly as this is not an examination or a test.

**SECTION A**

**Instructions:** Please tick (√) the most appropriate option or fill in your response.

1. Age: …….... years

2. Ethnicity

- Kanuri
- Hausa
- Babur
- Shuwa
- Marghi
- Fulani
- Others (Specify) ……………….

3. Educational status

- No formal educational
- Primary
- Secondary
- Tertiary

4. Occupation

- None/housewife
- Self-employed
- Government employed
- Private employment
- Student

5. Residence in Maiduguri

- Permanent Resident
- Internally displaced person

**SECTION B (INFORMATION)**

**Instructions:** Please tick (√) the most appropriate option based on the options provided which are: **YES**, **NO** or **I DON’T KNOW** for each of the statements below.

| **S/NO** | **Questions** | **Response** | | |
| --- | --- | --- | --- | --- |
|  |  | **YES** | **NO** | **I DON’T KNOW** |
| 1 | Malaria is transmitted through mosquito bite |  |  |  |
| 2 | Does pregnancy increase the chances of contracting malaria? |  |  |  |
| 3 | Does malaria have harmful effects on the pregnant woman? |  |  |  |
| 4 | Does malaria have harmful effects on the foetus? |  |  |  |
| 5 | Malaria can cause the pregnant woman to have insufficient blood |  |  |  |
| 6 | Malaria can cause miscarriage |  |  |  |
| 7 | Malaria can cause pre-term delivery |  |  |  |
| 8 | Malaria can cause can cause the baby to have a low birth weight |  |  |  |
| 9 | Malaria can cause death of the mother |  |  |  |
| 10 | Malaria can cause death of the foetus |  |  |  |
| 11 | Insecticidal nets are used to keep mosquitoes away |  |  |  |
| 12 | Insecticidal nets are more effective compared to plain nets |  |  |  |
| 13 | The chemicals on Insecticidal nets can be dangerous to one who sleeps under it |  |  |  |
| 14 | Insecticidal nets should be washed after every one month |  |  |  |
| 15 | Insecticidal nets should be washed after every 3-4 months |  |  |  |
| 16 | Insecticidal nets should be washed with water and ordinary soap only |  |  |  |
| 17 | Insecticidal nets should be washed with water and detergent |  |  |  |
| 18 | Insecticidal nets should be dried under the shade |  |  |  |

**SECTION C (MOTIVATION)**

**Instructions:** Please TICK (√) only one answer for each statement. Each statement is answered based on a 5-point scale which ranges from 1 = very bad to 5 = very good; or from 1 = very unpleasant to 5 = very pleasant.

| a | **Please tell us how good or bad the following are for your health** | **very bad** | **somewhat bad** | **neither bad nor good** | **somewhat good** | **very good** |
| --- | --- | --- | --- | --- | --- | --- |
| MOT1 | For the remaining duration of your pregnancy, how good or bad would it be for your health to sleep more frequently under an insecticidal net? |  |  |  |  |  |
| **b** | **Please tell us how pleasant or unpleasant it would be for you to do the behaviour** | **very unpleasant** | **somewhat pleasant** | **neither unpleasant nor pleasant** | **somewhat pleasant** | **very pleasant** |
| MOT2 | For the remaining duration of your pregnancy, how pleasant or unpleasant would it be for you to sleep more frequently under an insecticidal net? |  |  |  |  |  |

**Instructions:** Please TICK (√) only one answer for each statement. Each statement is answered based on a 6-point scale which is:

1: Very untrue

2: Mostly untrue

3: Untrue

4: True

5: Mostly true

6: Very true

|  | **Please tell us how true or untrue it is for you** | **very untrue** | **mostly untrue** | **untrue** | **true** | **mostly true** | **very true** |
| --- | --- | --- | --- | --- | --- | --- | --- |
| MOT3 | Most people who are important to me think I should sleep more frequently under an insecticidal net |  |  |  |  |  |  |

**SECTION D (BEHAVIOURAL SKILLS)**

**Instructions:** This section asks about level of difficulty/ease and effectiveness/ineffectiveness in performing certain tasks.

Please TICK (√) only one answer for each statement. Each statement is answered according to a four point scale which is:

1: Very hard

2: Hard

3: Easy

4: Very easy

| **a** | **Right now, how easy or hard would it be for you to…** | **Very hard**  **1** | **Hard**  **2** | **Easy**  **3** | **Very easy**  **4** |
| --- | --- | --- | --- | --- | --- |
| B_SKILLS_1 | Sleep under an insecticidal bed net every night |  |  |  |  |
| **b** | **Right now, how effectively or ineffectively could you…** | **Very Ineffectively**  **1** | **Ineffectively**  **2** | **Effectively**  **3** | **Very Effectively**  **4** |
| B_SKILLS_2 | Properly hang your insecticidal net? |  |  |  |  |
| B_SKILLS_3 | Check for and repair holes and rifts in your insecticidal bed net? |  |  |  |  |
| B_SKILLS_4 | Sleep more frequently under an insecticidal bed net? |  |  |  |  |
| B_SKILLS_5 | Persuade others to support your sleeping under an insecticidal bed net? |  |  |  |  |

**SECTION E (BEHAVIOUR)**

**Instructions:** This section asks about how frequently you practice certain tasks.

Please TICK (√) only one answer for each statement. Each statement is answered according to a five point scale which is:

0 times a week: Never

1-2 times a week: Seldom

3-4 times a week: Sometimes

5-6 times a week: Often

7 times a week: Almost always

|  | **STATEMENT** | **SCALE** | | | | |
| --- | --- | --- | --- | --- | --- | --- |
| **S/N** |  | **Never** | **Seldom** | **Sometimes** | **Often** | **Almost always** |
| BEHAVIOUR | How often do you sleep under an Insecticidal net? |  |  |  |  |  |
